# Scalable Copper Sulfide Formulations for Super‐Resolution Optoacoustic Brain Imaging in the Second Near‐Infrared Window

**DOI:** 10.1002/smtd.202400927

**Published:** 2024-10-24

**Authors:** Lin Tang, Daniil Nozdriukhin, Sandeep Kumar Kalva, Quanyu Zhou, Çağla Özsoy, Shuxin Lyu, Michael Reiss, Anxo Vidal, Ana Torres, Xosé Luís Deán‐Ben, Daniel Razansky

**Affiliations:** ^1^ Institute of Pharmacology and Toxicology and Institute for Biomedical Engineering Faculty of Medicine University of Zurich Zurich 8057 Switzerland; ^2^ Institute for Biomedical Engineering Department of Information Technology and Electrical Engineering ETH Zurich Zurich 8093 Switzerland; ^3^ Department of Biosciences and Bioengineering Indian Institute of Technology Bombay Mumbai 400076 India; ^4^ Department of Medical Imaging Shanxi Medical University Taiyuan 030001 China; ^5^ Center for Research in Molecular Medicine and Chronic Diseases (CiMUS) and Health Research Institute of Santiago de Compostela (IDIS) University of Santiago de Compostela Santiago de Compostela 15782 Spain; ^6^ Experimental Biomedicine Centre (CEBEGA) University of Santiago de Compostela Santiago de Compostela 15782 Spain; ^7^ Zurich Neuroscience Center (ZNZ) Zurich 8057 Switzerland

**Keywords:** chemical imaging, microparticle localization, neuroimaging, optoacoustic imaging, photoacoustics, second near‐infrared window

## Abstract

Optoacoustic imaging offers label‐free multi‐parametric characterization of cerebrovascular morphology and hemodynamics at depths and spatiotemporal resolution unattainable with optical microscopy. Effective imaging depth can greatly be enhanced by employing photons in the second near‐infrared (NIR‐II) window. However, diminished absorption by hemoglobin along with a lack of suitable contrast agents hinder an efficient application of the technique in this spectral range. Herein, copper sulfide (CuS) micro‐ and nano‐formulations for multi‐scale optoacoustic imaging in the NIR‐II window are introduced. Dynamic contrast enhancement induced by intravenously administered CuS nanoparticles facilitated visualization of blood perfusion in murine cerebrovascular networks. The individual calcium carbonate microparticles carrying CuS are further shown to generate sufficient responses to enable super‐resolution microvascular imaging and blood flow velocity mapping with localization optoacoustic tomography.

## Graphical abstract

This study introduces CuS‐loaded polydopamine‐coated CaCO_3_ microparticles (CaCO_3_@CuS/PDA MPs) combined with localization optoacoustic tomography (LOT) for high‐resolution imaging of brain microvasculature and precise mapping of blood flow velocity in the NIR‐II window, overcoming the resolution limits imposed by acoustic diffraction. The new contrast agent enhances optoacoustic imaging capabilities and facilitates studies into vascular‐related pathologies.

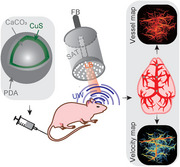

## 1 Introduction

Cerebrovascular system plays a pivotal role in maintaining physiological brain function and is closely associated with a myriad of neurological disorders. Alterations in vascular morphology, topology, and function are widely recognized as key factors of Alzheimer's disease (AD), acute ischemic stroke (AIS), and other neurological disorders.^[^ ^1^, ^2^ ^]^ Early diagnosis of these conditions and effective therapeutic monitoring can greatly benefit from new imaging methods capable of multiparametric characterization of cerebrovascular morphology and hemodynamics at multiple spatial and temporal scales.

Optoacoustic (OA) imaging is a non‐invasive and label‐free modality capable of deep tissue imaging of optical absorption contrast with high spatial resolution, owing to its insensitivity to photon scattering within biological tissues.^[^ ^3^, ^4^, ^5^ ^]^ The spectroscopic selectivity of OA facilitates its unique in vivo chemical imaging performance to quantify the biodistribution of intrinsic molecules and extrinsically administered substances.^[^ ^6^, ^7^, ^8^ ^]^ State‐of‐the‐art OA tomography systems can attain spatial resolution in the 100 µm range,^[^ ^9^, ^10^, ^11^ ^]^ volumetric imaging rates of hundreds to thousands of frames per second,^[^ ^12^, ^13^, ^14^ ^]^ as well as real‐time multi‐spectral (multi‐wavelength) imaging performance,^[^ ^15^ ^]^ effectively enabling 5D visualization along the spatial, temporal, and spectral dimensions simultaneously. Imaging depth of millimeters to centimeters into biological tissues is achievable, well beyond what is possible with optical microscopy.^[^ ^16^ ^]^ Specific spectral signatures of hemoglobin in oxygenated and deoxygenated states across the visible and near‐infrared (NIR) ranges further allow for label‐free visualization of blood vessel morphology with simultaneous mapping of the oxygen saturation.^[^ ^17^, ^18^ ^]^

Tissue excitation with NIR optical wavelengths is generally preferred for OA imaging to maximize light penetration into biological tissues.^[^ ^19^ ^]^ A particularly important range is the so‐called second near‐infrared (NIR‐II) window (1000–1700 nm), where 1) cost‐effective short‐pulsed solid‐state lasers with high per‐pulse energy are widely available; 2) light penetration is maximized due to reduced optical absorption and scattering; 3) the permissible laser energy deposition conforming to the international laser safety standards is maximized.^[^ ^20^ ^]^ On the other hand, employing the NIR‐II wavelength range comes to the detriment of diminished hemoglobin absorbance, hence reducing angiographic contrast as compared to the visible and NIR‐I wavelength ranges.

While the high temporal resolution of OA tomography facilitates visualization of an intravenously injected bolus to assess tissue perfusion, it has recently been shown that individual detection and tracking of micron‐sized absorbing particles further allows breaking through the spatial resolution limits imposed by acoustic diffraction.^[^ ^21^, ^22^ ^]^ The so‐called localization optoacoustic tomography (LOT) has employed extremely absorbing (manifold higher absorption cross‐section than red blood cells) microdroplets to achieve capillary‐level‐resolution imaging in the mouse model of acute ischemic stroke, further enabling quantitative measurements of blood flow velocity.^[^ ^22^ ^]^ However, only a relatively sparse distribution of liquid droplets could be used due to toxicity concerns and potential aggregation, resulting in prolonged acquisition times for rendering a super‐resolution image.

Despite the wide availability of absorbing dyes and nanoparticles, there is a lack of contrast agents optimized for OA imaging in the NIR‐II window, particularly when considering the emergence of LOT as a microparticle‐based super‐resolution imaging approach. Vaterite‐type calcium carbonate (CaCO_3_) particles have received growing attention in biomedicine due to their porous structure, biocompatibility, biodegradability, tunable size, and low cost.^[^ ^23^ ^]^ Various bioactive substances, such as doxorubicin and catalase, have been loaded into vaterite by physical adsorption or co‐precipitation. The suitable size and high carrying capacity of CaCO_3_ microparticles (MPs) make them attractive to become excellent component for synthesizing LOT contrast agents. Copper sulfide nanoparticles (CuS NPs) exhibit strong NIR‐II absorbance and have been shown to enhance OA imaging contrast and penetration depth.^[^ ^24^ ^]^ Given their ultrasmall size (≈10 nm), they can easily be cleared from the body through metabolism^[^ ^25^ ^]^ and potentially be loaded into microparticulate core templates. Polydopamine (PDA), a light‐absorbing polymer formed through oxidative polymerization of dopamine under alkaline conditions, has been exploited as a biocompatible and biodegradable material for imaging and photothermal therapy purposes.^[^ ^26^, ^27^ ^]^Moreover, PDA can be readily deposited onto the surface of a wide range of inorganic and organic materials to optimize and enhance their functionalities.^[^ ^28^ ^]^

Here we introduce a universal platform for in vivo multi‐scale OA vascular imaging in the NIR‐II window based on CuS micro‐ and nano‐particulate formulations. Dynamic NIR‐II contrast enhancement induced by intravenously administered pegylated CuS NPs is shown to enable deep tissue contrast enhancement and visualization of blood perfusion in cerebrovascular networks in mice. In addition, CuS‐loaded PDA‐coated CaCO_3_ MPs are shown to seamlessly flow through the bloodstream and generate sparsely distributed responses within the sensitivity range of LOT. Accurate particle tracking thus enables microvascular imaging and blood flow velocity mapping with superb sensitivity and spatial resolution.

## 2 Results

### 2.1 Synthesis of the Scalable Copper Sulfide Formulations

**Figure 1:**
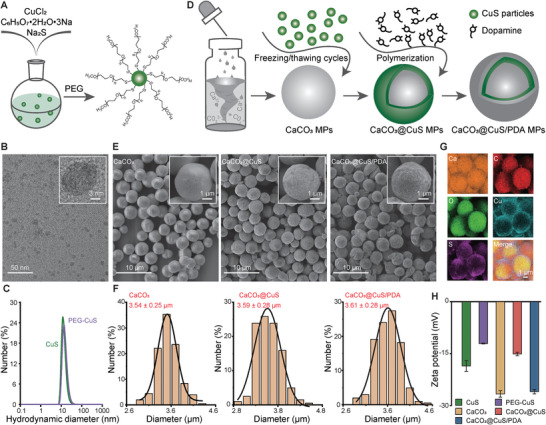
Synthesis of CuS NPs and CaCO_3_@CuS/PDA MPs. A) Schematic representation of CuS NPs synthesis. B) TEM image of CuS‐PEG NPs and an HR‐TEM microphotograph of solitary nanoparticle with visible crystalline planes in the inset. C) Size distribution of CuS and PEG‐CuS particles measured with DLS. D) Schematic illustration of the preparation process for CaCO_3_@CuS/PDA MPs. E) SEM images and F) corresponding size distribution with Gaussian fitting for CaCO_3_ MPs, CaCO_3_@CuS MPs, and CaCO_3_@CuS/PDA MPs, respectively. G) Distribution of elements on the CaCO_3_@CuS/PDA MPs surface acquired with EDS. H) Zeta‐potential of all the particle types used in the manuscript (*n* = 3). Data was shown as mean ± standard deviation (SD).

Multi‐scale CuS‐based particles were synthesized to provide OA contrast in the NIR‐II window. Citrate‐stabilized CuS NPs were initially prepared with a modified version of a reported method,^[^ ^29^ ^]^ followed by ligand exchange with thiolated polyethylene glycol (MeO‐PEG‐SH) (**Figure** **1**A, see Experimental Section for details). Successful nanoparticle synthesis was confirmed with transmission electron microscopy (TEM), demonstrating diameter distributions of 9.08 ± 3.32 nm for pristine (CuS) and 10.73 ± 3.34 nm for PEGylated NPs (PEG‐CuS), whereas dynamic light scattering (DLS) measurement revealed 13.13 ± 6.16 nm and 14.30 ± 1.14 nm size distributions for pristine and PEGylated NPs, respectively (Figure 1B,C). The discrepancy could be attributed to partial aggregation of uncoated CuS NPs during the TEM sample preparation or excellent dispersity of CuS NPs after PEGylation (Figure S1, Supporting Information). NPs were progressively deposited onto the surface of CaCO_3_ MPs using freeze‐loading technique (CaCO_3_@CuS MPs)^[^ ^30^ ^]^, and an additional PDA layer based on the self‐polymerization of dopamine in Tris buffer of pH 8 was deposited to modify the MPs surface (CaCO_3_@CuS/PDA MPs) (Figure 1D). Scanning electron microcopy (SEM) and TEM images of the CaCO_3_ particles revealed their spherical shape with a spherulitic structure, corroborating successful formation of cell‐sized vaterite polymorphs (Figure 1E; Figure S2, Supporting Information). The CaCO_3_@CuS and CaCO_3_@CuS/PDA particles exhibited sustained spherical morphology and relatively uniform size, with visual differences in surface topology and increased size associated with CuS loading (35.5 ± 8.9 nm) and PDA coating (22.5 ± 0.9 nm) (Figure 1E; Figure S2, Supporting Information). Specifically, diameter distributions of 3.54 ± 0.25, 3.59 ± 0.28, and 3.61 ± 0.28 µm were calculated from SEM images for CaCO_3_, CaCO_3_@CuS, and CaCO_3_@CuS/PDA, respectively (Figure 1F). Energy‐dispersive X‐ray spectroscopy (EDS) mapping confirmed distributed signals from copper and sulfur on the surface of CaCO_3_ MPs and increased contents of carbon and oxygen after PDA coating, signifying the formation of CaCO_3_@CuS/PDA (Figure 1G; Figure S3, Supporting Information). Inductively coupled plasma optical emission spectroscopy (ICP‐OES) measurements and standard curve of CuS NPs further demonstrated a loading capacity up to 84.6 ± 3.6% from the stock solution (Figure S4A, Supporting Information). The measured zeta potentials of CuS, PEG‐CuS, CaCO_3_, CaCO_3_@CuS, and CaCO_3_@CuS/PDA were also consistent with functionalization and chemical loading (Figure 1H). The PEG chains covering the CuS NPs partially shield the negative charge of these, leading to an increase in zeta potential (from −18.6 ± 1.53 to −12.13 ± 0.12 mV). On the other hand, the increase of MPs zeta‐potential from −26.67 ± 0.93 to −15.13 ± 0.49 mV after the CuS deposition is consistent with NPs covering of the surface, while a subsequent decrease down to −26 ± 0.62 mV for CaCO_3_@CuS/PDA is associated to the negative PDA charge. When deionized (DI) water was replaced by PBS and DMEM, the zeta potential values were less negative (Figure S4B, Supporting Information). However, despite this change, the MPs suspended in different physiological media maintained the initial size, morphology, and good dispersibility throughout one‐week observation period (Figure S5, Supporting Information).

### 2.2 Visualization of CuS Nanoparticle Perfusion in the NIR‐II Optical Window

**Figure 2:**
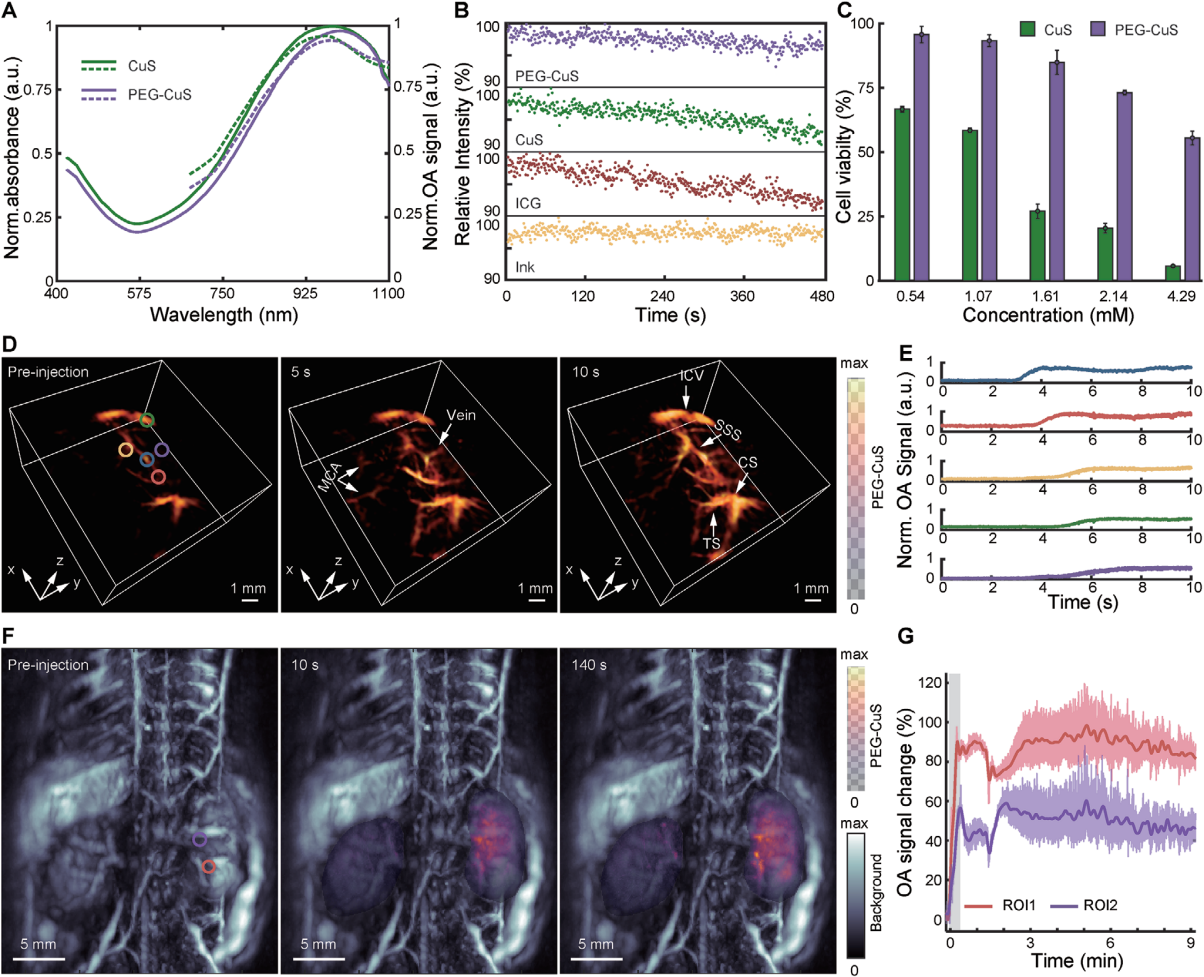
Optoacoustic tomography imaging with CuS NPs in NIR‐II window. A) UV–vis–NIR extinction and OA absorption spectra of CuS and PEG‐CuS NPs. B) The evolution of OA signal intensity of CuS‐based materials depending on the laser illumination time (1064 nm as excitation wavelength: ink, CuS, and PEG‐CuS NPs; 797 nm as excitation wavelength: ICG). C) AlamarBlue cell viability assay results for CuS and PEG‐CuS NP (*n* = 3). D) 3D‐view of mouse brain vasculature before, during, and after the injection of PEG‐CuS NPs (36.35 mм, 100 µL). MCA – middle cerebral artery, ICV – inferior cerebral veins, SSS – superior sagittal sinus, TS ‐transverse sinus, CS – confluence of sinuses. E) OA signal changes observed within the defined regions marked by colored circles in panel D. F) Optoacoustic images of murine kidney region acquired before, after 10 s, and after 140 s PEG‐CuS NPs administration. G) Kidney region OA signal change observed within the defined regions marked by colored circles in panel F. Data was presented as the mean ± SD.

Blood pool contrast agents are routinely used in biomedical imaging to increase the visibility of cardiovascular structures.^[^ ^31^ ^]^ A desired property of these chemical substances is long circulation time. In principle, OA can visualize blood vessels in a label‐free manner by capitalizing on the strong endogenous absorption of hemoglobin in the visible and the first near‐infrared spectral window but to the detriment of penetration depth. The synthesized CuS and PEG‐CuS NPs exhibit light absorption extending into the NIR‐II range, where light attenuation and background OA contrast are minimized due to reduced scattering and absorption by hemoglobin and water (**Figure** **2**A). Repeated exposure of PEG‐CuS NPs to 1064 nm laser pulses with an optical fluence of ≈24.2 mJ cm^−2^ resulted in an OA signal decay similar to that observed for indocyanine green (ICG) when exposed to approximately the same energy density at its 797 nm peak absorption wavelength (Figure 2B, ≈10% decay after 4800 laser pulses). This could either be attributed to partial morphological changes of the particles or their chemical decomposition.^[^ ^32^, ^33^ ^]^

PEGylation was shown to improve photostability of the particles (≈5% decay after 4800 laser pulses), arguably due to the blocking of oxygen migration toward their surface and further surface photobleaching.^[^ ^34^, ^35^ ^]^ PEG‐CuS were also shown to be less toxic to Chinese Hamster Ovarian (CHO) cells than pristine CuS for the same concentration (Figure 2C, see Experimental Section for details). Indeed, cell viability after exposure to PEG‐CuS was notably high, even for nanoparticle concentrations resulting in strong OA signal generation. Real‐time OA imaging of the murine brain at 1064 nm during intravenous injection of PEG‐CuS (100 µL, 36.35 mм) suspended in phosphate‐buffered saline (PBS) led to a clearly enhanced angiographic contrast (Figure 2D). Cortical and subcortical vessels, not visible in the baseline OA image, could be resolved post‐injection with PEG‐CuS‐based contrast. Particularly, the middle cerebral artery (MCA) and the veins became discernible following the PEG‐CuS injection.

The high temporal resolution (200 Hz) of the tomographic OA imaging system further facilitated the assessment of the vessel perfusion. Time delays between the bolus appearance at selected vessels were quantified (Figure 2E), providing a valuable metric for the detection of microcirculatory alterations. A more comprehensive visualization of the of PEG‐CuS particle perfusion is provided in a movie available in the online version of the journal (Movie S1, Supporting Information). Kidney perfusion dynamics of the particles were further assessed by scanning the entire dorsal area of the mouse with a single‐sweep volumetric optoacoustic tomography (sSVOT) system.^[^ ^36^ ^]^ An enhanced OA signal was observed across the kidney after intravenous injection of NPs (Figure 2F), with no obvious differences between temporal profiles of the OA signals in different regions (Figure 2G). This observation substantiates the assertion that PEG‐CuS NPs do not undergo renal clearance, as differential contrast agent kinetics for the renal artery, cortex, and pelvis would be expected in such instances.

### 2.3 Fast Super‐Resolution Optoacoustic Imaging with CuS Microparticles

**Figure 3:**
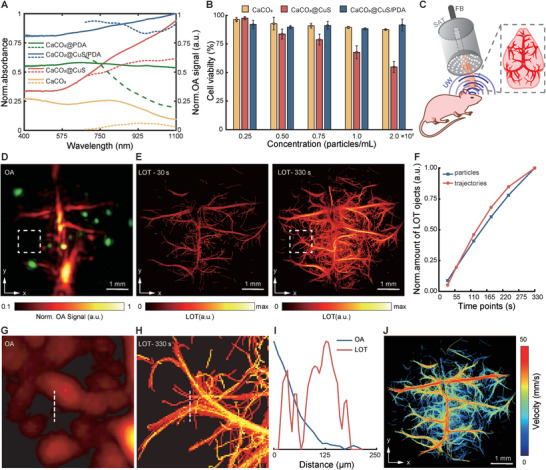
Localization Optoacoustic Tomography (LOT) of the mouse brain in the NIR‐II window. A) UV—vis–NIR extinction spectra of CaCO_3_‐based microparticles measured with a spectrophotometer (solid lines) along with their corresponding OA spectra in the NIR range (dotted lines). B) Cell toxicity of coated and uncoated microparticles performed on CHO cell culture using alamarBlue assay (*n* = 3). C) The principal scheme of the OA imaging of the mouse brain. FB – fiber bundle, SAT – spherical array transducer, LB – light beam, UW – ultrasound waves. D) The reconstructed OA image of the mouse brain after injecting the CaCO_3_@CuS/PDA MPs: red color denotes cerebral vasculature, green spheroids – signals from the microparticles. E) LOT images reconstructed in 30 and 330 s time frames post‐injection: after 30 s major vessels appeared and after 330 s fine microvasculature could be resolved. F) Normalized amount of localized objects depending on the reconstruction time frame. G) Conventional OA reconstruction within a region lacking green spheroids (white frame in panel D) prior to injecting the CaCO_3_@CuS/PDA MPs. H) The corresponding LOT image of the region within the white frame in panel E. I) 1D line profiles of the OA and LOT images along the dotted lines in panels G and H. J) The blood flow velocity map of the mouse brain reconstructed via particle tracking over time. Data was shown as mean ± SD.

The spatial resolution of OA imaging at depths beyond the diffusive limit of light (≈1 mm in biological tissues) is fundamentally restricted by the effective ultrasound detection bandwidth, namely, the acoustic diffraction limit.^[^ ^37^ ^]^ Recently, localization and tracking of intravenously injected MPs have enabled overcoming this barrier to achieve super‐resolved functional microangiographic imaging.^[^ ^22^, ^38^ ^]^ For this, particles with strong per‐unit absorptivity to be individually detected and distinguishable against the blood background whilst being sufficiently small to circulate through capillary networks are needed. The synthesized CaCO_3_@CuS/PDA MPs (3.61 ± 0.28 µm diameter) were smaller than murine red blood cells (≈6 µm) and exhibited strong optical absorption properties in the NIR‐II window attributed to the CuS NPs loading and PDA coating (**Figure** **3**A). The OA signal strength was shown to exhibit a linear increase with MP concentration (Figure S6A, Supporting Information). Repeated exposure of CaCO_3_@CuS/PDA to 1064 nm laser pulses with an optical fluence of ≈24.2 mJ cm^−2^ resulted in a stable OA signal as compared to a notable signal decay from CaCO_3_@CuS exposed to the same energy density (Figure S6B, Supporting Information). Note that the excitation light source does not significantly trigger the release of CuS NPs from MPs (Figure S7, Supporting Information). The CHO cell viability assays demonstrated that the PDA coating improves the biocompatibility of CaCO_3_@CuS/PDA compared to CaCO_3_@CuS (Figure 3B, see Experimental Section for details).

LOT imaging of the mouse brain was based on high‐frame‐rate volumetric detection of individual CaCO_3_@CuS/PDA MPs with a spherical array transducer (Figure 3C). Singular value decomposition (SVD) filtering of a sequence of images enabled differentiating the slowly changing background signal generated by vessels from the fast‐moving spheroids corresponding to MPs circulating in the bloodstream (red and green channels in Figure 3D, respectively). The high density of the MPs enabled the building of a detailed microvascular map by superimposing their localized positions within a relatively low number of consecutive frames (Figure 3E). In contrast, LOT imaging based on previously reported microdroplets necessitated a substantially larger number of frames, as high concentrations could not be employed due to toxicity concerns.^[^ ^22^ ^]^ A rotating view of the LOT image rendered with all frames is available in the online version of the journal (Movie S2, Supporting Information). The assessment of imaging performance involved analyzing the number of localized CaCO_3_@CuS/PDA particles and trajectories generated within a specified time or frame count (Figure 3F). The quantitative analysis encompassed the assessment of vessel percentage area (VPA) and total number of junctions (TNJ) to further evaluate the performance of LOT in relation to recording time (Figure S8, Supporting Information). The super‐resolution imaging capacity becomes evident when comparing selected regions and profiles of the regular OA versus LOT images in the transverse, sagittal, and coronal planes (Figure 3G–I; Figure S9, Supporting Information). Microvascular structures corresponding to small arterioles, venules, and penetrating vessels are readily resolved with LOT, whilst only large vessels are visible in the standard OA image. Notably, penetrating vessels at ≈1.9 mm depth turned clearly distinguishable at ≈20 µm resolution. Importantly, the high‐frame‐rate tracking of particles enables quantifying blood flow velocity in the mouse brain, reaching up to ≈50 mm s^−1^.^[^ ^22^ ^]^ A blood velocity map rendered with LOT in the transverse plane revealed the expected higher blood flow velocity in major vessels gradually decreasing along their branching pathways (Figure 3J).

### 2.4 Pharmacokinetics and Biosafety of Micro‐ and Nano‐Formulations

**Figure 4:**
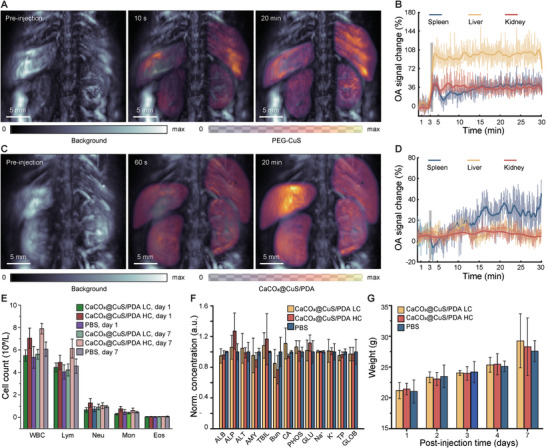
Pharmacokinetics, biodistribution, and biosafety of PEG‐CuS NPs and CaCO_3_@CuS/PDA MPs in mice. A) Coronal maximum intensity projections (MIPs) NPs showing a signal increase of at 20 min post‐injection compared to pre‐injection. B) Volumetric signal changes (baseline subtracted), measured across the entire liver, spleen, and kidneys of a live mouse, following injection of NPs. C) Coronal MIPs MPs showing a signal increase of at 20 min post‐injection compared to pre‐injection. D) Volumetric signal changes (baseline subtracted), measured across the entire liver, spleen, and kidneys of a living mouse, following injection of MPs. E) The results of hematology at 1 and 7 days post‐injection of CaCO_3_@CuS/PDA MPs at low and high concentrations in comparison to injection of PBS (*n* = 4). F) Blood biochemistry at 7 days post injection of CaCO_3_@CuS/PDA MPs in comparison to PBS injection. ALB – albumin, ALP – alkaline phosphatase, ALT – alanine transaminase, AMY – amylase, TBIL – total bilirubin, BUN – blood urea nitrogen, CA – calcium, PHOS – phosphates, GLU – glutamine, Na^+^ – sodium, K^+^ – potassium, TP – total protein, GLOB – globulin. G) Weight development of mice injected with CaCO_3_@CuS/PDA MPs at low concentration, high concentration and PBS within 7 days post‐administration. Data was presented as mean ± SD.

The body's reaction to intravenously administered particulate agents is influenced by an array of factors, encompassing size and shape, composition, as well as surface chemistry.^[^ ^39^ ^]^ The safe application of PEG‐CuS NPs has previously been evaluated.^[^ ^25^, ^40^ ^]^ Their hydrodynamic diameter is small enough to hamper rapid recognition and uptake by phagocytic cells yet sufficiently large due to adequate PEGylation to prevent fast kidney clearance (Figure 2F,G), which results in prolonged circulation time. in vivo images of the biodistribution of i.v. injected PEG‐CuS NPs recorded with sSVOT were in alignment with this expectation (**Figure** **4**A, see Experimental Section for details). Specifically, a strong rise of the OA signal in the kidneys, spleen, and liver has been observed shortly after administration of a PEG‐CuS suspension in PBS (100 µL bolus, 36.35 mм), and sustained for a relatively long duration (Figure 4B; Figure S10A,B, Supporting Information). On the other hand, time‐resolved images of the in vivo biodistribution of CaCO_3_@CuS/PDA MPs exhibited a fundamentally different pattern (Figure 4C). No significant OA signal changes were noted in selected organs immediately after injecting the MP suspension in PBS (100 µL bolus, 2 × 10^8^ particles mL^−1^), arguably due to their sparse distribution inside the blood pool (Figure 4D; Figure S10C,D, Supporting Information). The OA signal in the spleen then progressively increased 10 min after injection, indicating MP accumulation in this organ. The observed reduced circulation time with respect to PEG‐CuS is generally expected considering the critical role of particle size in the interaction with the reticuloendothelial system (RES).

Intravenous injection of micron‐size objects also raises concerns about microvascular blockage induced by aggregation of particles and consequent organ failure, as well as other adverse effects associated with the novel formulation. Thereby, we systematically analyzed the newly developed CaCO_3_@CuS/PDA MPs for any potential toxic effects. Capillary blockage was not detected within the LOT image sequence (Figure 3D; Movie S3, Supporting Information). This is consistent with the fact that no aggregation of particles was visible in histological sections of the brain and other organs from mice euthanized post i.v. injection (Figure S11, Supporting Information). The acute toxicity of CaCO_3_@CuS/PDA particles was further assessed by monitoring the condition of three groups of mice for one week following administration of a 100 µL bolus of 1) CaCO_3_@CuS/PDA at a concentration of 2 × 10^8^ particles mL^−1^ (referred as HC), 2) CaCO_3_@CuS/PDA at a concentration of 5 × 10^7^ particles mL^−1^ (referred as LC), and 3) PBS only (see Experimental Section for details). No significant physical or behavioral differences were observed in mice scored for a 7‐day period following injection. Similar hemodynamic parameters were also observed in all groups when measured on days 1 and 7 post‐injection (Figure 4E). A more detailed biochemical analysis was performed post‐euthanasia, further revealing no notable differences across the three groups thus excluding the possibility of kidney or liver failure (Figure 4F). The weight of mice injected with MPs harmonically increased over a one‐week period without significant deviation from the control group (Figure 4G).

## 3 Discussion

The emerging field of chemical imaging can largely benefit from new imaging modalities having sufficient spatial, temporal, and/or spectral resolution to efficiently identify and map the biodistribution of bio‐chromes and biochemical processes occurring inside living organisms.^[^ ^8^ ^]^ Spatially resolved dynamic chemical mapping can play a pivotal role in identifying specific disease biomarkers while enabling a better understanding of how drugs are metabolized and distributed throughout the body, thus facilitating the development of more effective and safer therapeutic approaches. While the successful application of OA for in vivo spectroscopic imaging of chemicals has long been documented,^[^ ^6^ ^]^ the recent developments in OA methods and instrumentation further resulted in unique multi‐scale imaging capacities encompassing imaging of individual cells to entire organs with the same type of contrast.^[^ ^41^, ^42^, ^43^ ^]^ New substances consistently providing spectrally‐distinct contrast across different spatial and temporal scales can thus bolster the OA chemical imaging capabilities.^[^ ^44^, ^45^ ^]^

Absorption of light by hemoglobin along with intense photon scattering by cellular structures limits the achievable depth, which is maximized for NIR‐II wavelengths shorter than the prominent infrared water absorption peak at around 1400 nm. Of particular importance is the fundamental wavelength emitted by standard short‐pulsed Nd:YAG lasers (1064 nm), which conveniently lies in a spectral gorge of water absorption after the first prominent peak. This fact, along with the high per‐pulse energy provided by these laser sources and the higher limit of safe exposure of skin to laser radiation, makes the 1064 nm wavelength a natural choice for OA imaging in the NIR‐II window.^[^ ^46^ ^]^ Small NPs with sizes ≈10 nm are optimal for achieving prolonged circulation, outperforming small dyes and larger NPs that are usually rapidly cleared through the kidneys or RES. PEGylation further increases particle solubility in water, prevents the protein corona formation in biological liquids, and hinders their recognition by macrophages, further prolonging the circulation time.^[^ ^47^, ^48^ ^]^ We have demonstrated that PEG‐CuS NPs exhibit prolonged circulation in the bloodstream, rendering them especially well‐suited as a blood‐pool agent for NIR‐II OA imaging. Additionally, our studies have shown that employing dynamic OA imaging during the injection of a bolus of CuS NPs allows for clear visualization of blood vessel perfusion, which can be used to assess complications associated with organ failure or tissue damage. The high frame rate of the tomographic OA system facilitated quantifying perfusion over brief intervals, rendering it well‐suited for assessing rapid changes, such as those induced by vasodilation or vasoconstriction.

The use of micron‐sized agents has previously resulted in unprecedented super‐resolution imaging performance with ultrasound localization microscopy (ULM).^[^ ^49^, ^50^ ^]^ Individual detection of circulating microbubbles enabled depicting vascular networks down to the capillary level, while particle tracking further enabled blood velocity mapping.^[^ ^49^ ^]^ Fluorescent MPs have similarly been used for microangiographic imaging from a sequence of wide‐field microscopy images.^[^ ^51^ ^]^ in vivo LOT imaging has been significantly challenged by the high background absorption of the red blood cells,^[^ ^52^ ^]^ which has partially been overcome by the use of microdroplets encapsulating a highly absorbing dye at two orders of magnitude higher concentration than hemoglobin in whole blood.^[^ ^22^ ^]^ Much like ULM, LOT enables microangiographic imaging beyond the acoustic diffraction barrier, while additionally providing oxygen saturation readings in the super‐resolved vessels along with the video‐rate 3D imaging capacity not easily realizable with ULM. These advanced microvascular imaging capabilities are expected to result in substantial progress in biomedical research. Potential applications include characterization of tumor microenvironment, assessment of coronary microcirculation, or quantification of hemodynamic changes associated to neuronal activity. The primary constraint of LOT was associated with the relatively sparse distribution of droplets that could be injected in vivo. Good quality microangiographic reconstructions could only be achieved after accumulating ≈20 000 frames, that is, over ≈200 s when imaging at 100 frames per second. Herein, we have shown that the newly introduced solid particles can be intravenously injected at a much higher concentration without leading to toxic effects in mice. This facilitated LOT imaging of the mouse brain with significantly higher effective temporal resolution and improved microangiographic image quality.

In conclusion, this study demonstrates the potential of CuS‐based nano‐ and micro‐formulations to significantly enhance the OA imaging performance in the NIR‐II window. In particular, limitations in vascular imaging associated with reduced hemoglobin absorption in this wavelength range and the fundamental resolution limit imposed by acoustic diffraction could be overcome. By leveraging the dynamic contrast properties of PEG‐CuS NPs, detailed visualization of the vessel morphology and dynamics across murine cerebrovascular networks was demonstrated. In addition, the individually detectable CaCO_3_@CuS/PDA MPs at the NIR‐II window prepared by a simple method facilitated super‐resolution imaging and precise blood flow velocity mapping with LOT. Our findings thus represent a significant advancement in the chemical imaging capabilities of OA, with promising applications aimed at a better understanding of vascular‐related pathologies. This progress will facilitate further research on neurological disorders by enhancing the extraction of significant signatures from the cerebrovascular system, thereby providing valuable insights into the formation, diagnosis, and treatment of brain diseases.

## 4 Experimental Section

### Materials

Copper chloride (CuCl_2_), sodium citrate tribasic dihydrate (C_6_H_5_O_7_·2H_2_O·3Na, SC), *O*‐(2‐Mercaptoethyl)‐*O′*‐methyl‐polyethylenglykol with a molecular weight of 2000 Da (MeO‐PEG‐SH), bovine serum albumin (BSA), sodium carbonate (Na_2_CO_3_), calcium chloride dihydrate (CaCl_2_·2H_2_O), dopamine hydrochloride (DA), ATX Tri buffer, agar, and Amicon Ultra‐15 centrifugal filter units with a 10 kDa molecular weight cut‐off were purchased from Sigma–Aldrich. Sodium sulfide (Na_2_S), Dulbecco's modified eagle medium (DMEM), fetal bovine serum (FBS), phosphate‐buffered saline (PBS), and alamarBlueTM cell viability reagent were purchased from Thermo Fisher Scientific. Indocyanine green (ICG) was purchased from TCI Deutschland GmbH. Chinese hamster ovarian (CHO) cell line was purchased from CLS Cell Lines Service GmbH. DI water with a resistivity of 18 MΩ cm^−1^ from a Mili‐Q water purification system (Millipore) was used in the experiments.

### Synthesis of CuS Nanoparticles

Citrate‐protected CuS NPs were synthesized following a modified version of the previously reported protocol.^[^ ^29^ ^]^ Briefly, CuCl_2_ (50 mм, 1 mL) and sodium citrate tribasic dihydrate (34 mм, 1 mL) were introduced to 48 mL of DI water in a round‐bottom flask under stirring at room temperature. After 5 min, Na_2_S (1 м, 100 µL) was added, resulting in a change of the mixture's color to dark brown. Subsequently, the mixture was transferred to an oil bath heated at 90 °C and stirred for 15 min until a dark‐green color emerged. Rapid cooling was achieved by submerging the flask in an ice‐water bath. The unreacted compounds were separated from the synthesized SC‐CuS NPs through 15 mL centrifugal filter units by centrifugation at 3350 RCF for 15 min using a Multifuge 1S‐R (Thermo Scientific, Germany). Finally, it was diluted to 5 mL with a concentration of 9.087 mм.

For PEGylation, the above CuS solution was added with 12 mg of MeO‐PEG‐SH and stirred overnight at a room temperature. Subsequently, the PEG‐CuS was washed three times with DI water and concentrated in a final volume of 1 mL.

### Synthesis of CaCO_3_ Microparticles Encapsulating CuS

The dispersion of CaCO_3_ MPs was prepared as follows. Na_2_CO_3_ (0.33 м, 0.5 mL) and CaCl_2_ (0.33 м, 0.5 mL) were added to an aqueous BSA solution (40 mg mL^−1^, 1 mL) under moderate stirring at 30 °C. Stirring was stopped after 30 s, allowing the MPs to ripe for 10 min. The resulting CaCO_3_ MPs were washed three times with DI water at 60 g RCF for 30 s (5415 D, Eppendorf, US) and subsequently dispersed in 1 mL of DI water. A mixture of 1 mL of CaCO_3_ MPs and 0.5 mL of CuS NPs in a 2 mL centrifuge tube was then rapidly refrigerated at −20 °C for 30 min to allow ice crystals to push NPs from the solution into the surface of MPs.^[^ ^30^ ^]^ Subsequently, the frozen samples were transferred to room temperature for complete thawing. After every two freezing/thawing cycles, the resulting suspension was collected into a 15 mL tube via centrifugation, and the resulting precipitate was redispersed in 1 mL of DI water for further loading. The above procedure was repeated until the total number of freeze/thaw cycles reached 14 times to achieve high loading efficiency.

The generated CuS‐loaded CaCO_3_ MPs (CaCO_3_@CuS) were subsequently dispersed in 0.5 mL of DI water and mixed with DA (4.35 mм, 1.5 mL) in Tris buffer under stirring at room temperature for 3 h to allow the DA to polymerize on the MPs surface. The resulting PDA‐coated CaCO_3_@CuS MPs (CaCO_3_@CuS/PDA) were washed and re‐suspended in 1 mL of PBS. The particle concentration was counted as 2.5 × 10^8^ particles mL^−1^, using a hemocytometer and a standard optical microscope (Primostar 3, ZEISS, Germany).

### Characterization of CuS‐Based Particles

The concentration of CuS NPs was calculated using inductively coupled plasma optical emission spectroscopy (ICP‐OES) (5100, Agilent Technologies, USA). 10 µL of prepared CuS NPs were added into 300 µL of fresh aqua regia in a rolled edge bottle (20 mL) for complete dissolution. The residual aqua regia was evaporated by heating at 80 °C. The resulting dry residue was then redissolved in 10 mL of 0.05 м HCl. By measuring spectral intensity at the Cu emission line 324.754 nm, the concentration of Cu was determined based on the established calibration curve of Cu standard solutions.

The NPs (CuS and PEG‐CuS) and MPs (CaCO_3_, CaCO_3_@CuS, and CaCO_3_@CuS/PDA) morphologies were studied using transmission electron microscopy (TEM). First, holey carbon film (Quantifoil R 2/1) on Cu 300 mesh coated with 2 nm of carbon was treated in a glow discharge system (Emitech K100X) for 45 s at 25 mA current. Then, this series of samples in an aqueous solution were drop‐casted on the grids and dried with the filter paper after 30 s of sedimentation. The remaining water was eliminated by drying the sample at room temperature for 1 h. The TEM grids were positioned in the sample holder and inserted into the JEOL JEM‐F200 HR‐TEM system. The microscopy images were acquired at 200 kV accelerating voltage and 20 µA electron gun emission using a Gatan Rio 16 camera at different magnification ratios. The data was processed in ImageJ 1.54 h.

Scanning Electron Microscopy (SEM) was used to assess the morphology and size of CaCO_3_ MPs. P‐doped silicon chips were washed in an ultrasonic bath with ethanol and DI water. 2 µL of each sample was subsequently drop‐casted on the chip and dried at room temperature. Samples were studied using Schottky field emission SEM Hitachi SU5000 in secondary electrons (SE) contrast at 3 kV accelerating voltage. Elemental composition was studied using Energy Dispersive Spectroscopy (EDS) with 10 kV accelerating voltage (Oxford Instruments Ultim Max detector).

The zeta potentials of the synthesized NPs and MPs were measured with a Zetasizer Nano‐ZS90 (Malvern Instrument, UK). The stock suspension of particles was injected into the folded capillary cell (DTS1070) and the zeta potential values were measured 3 times at 25 °C.

The UV–vis–NIR spectra were measured with a UV‐1900i spectrophotometer (Shimadzu, Japan). For that, particle suspension was located in the synthetic quartz glass cuvette (CV10Q7F, Thorlabs) and the absorption was measured in the 420–1100 nm wavelength range.

### Loading Efficiency of CaCO_3_@CuS/PDA

The concentration of CuS NPs in the suspension of MPs before and after freezing/thawing cycles was estimated by measuring absorbance at 997 nm, corresponding to a standard curve of CuS NPs. The curve exhibited a linear behavior with a squared correlation coefficient of 0.9996 within a range of 0.22–2.2 mм CuS concentrations in DI water. The loading efficiency of CuS NPs on MPs was determined as follows:

(1) $$\begin{array}{ccc} & & \mathrm{Loading}\;\mathrm{efficiency}\;\\ & & \;=\frac{\mathrm{Total}\;\mathrm{amount}\;\mathrm{of}\;\mathrm{CuS}\;\mathrm{NPs}-\mathrm{Free}\;\mathrm{CuS}\;\mathrm{NPs}\;\mathrm{in}\;\mathrm{the}\;\mathrm{supernatant}}{\mathrm{Total}\;\mathrm{amount}\;\mathrm{of}\;\mathrm{CuS}\;\mathrm{NPs}}\\ & & \;\times \;100\% \end{array}$$

### Colloidal Stability Assessment

Equal amounts of CaCO_3_@CuS/PDA MPs were dispersed in DI water, PBS, and DMEM to prepare stock solutions. The zeta potential values of MPs in different media were measured using Zetasizer Nano‐ZS90. The stock solutions were monitored over one week with photographs and bright‐field microscopy images (Primostar 3, ZEISS) connected to an area scan camera (a2A4504‐18umBAS, Basler).

### Optoacoustic Spectra and Photostability

The optical absorption spectra of the synthesized NPs and MPs were measured with a custom‐designed OA system. Signal excitation was done with a 100 Hz optical parametric oscillator (OPO)‐based laser (Model: SpitLight EVO II OPO‐532, Innolas GmbH, Krailling, Germany) over 680–1100 nm wavelength range. A 512‐element spherical array transducer with a central frequency of 7 MHz and >80% detection bandwidth was used for tomographic acquisition of the generated OA responses, which were subsequently digitized at 40 megasamples per second using a custom‐made data acquisition system (DAQ, Falkenstein Mikrosysteme GmbH, Taufkirchen, Germany) triggered with the Q‐switch output of the laser. 3D images were subsequently reconstructed using a graphics processing unit (GPU)‐based back‐projection algorithm.^[^ ^53^, ^54^ ^]^ Particle solutions were injected in a 500 µm inner diameter polyethylene tubing placed within the field of view (FOV) of the OA imaging system. An average signal value within the tubing was considered. Spectral curves representing OA signal intensity across different wavelengths were reconstructed after correcting for spatial and temporal fluence variations using the optical and OA spectra of ink as a reference. Using the same setup at 1064 nm, the correlation between the OA signal and MPs concentrations was determined. The photostability of the MPs and their components (CuS NPs, PEG‐CuS NPs, CaCO_3_@CuS MPs, and CaCO_3_@CuS/PDA MPs) were assessed with the same setup and compared to standard substances (ink and ICG). The measurements were conducted for 8 min at 1064 nm wavelength using laser fluence of ≈24.2 mJ cm^−2^ at the sample.

### Loading Stability of CuS NPs on MPs

A 1.5 mL dispersion of MPs was centrifuged at 60 g RCF for 30 s to collect the supernatant. The absorbance of the supernatant in the 400 −1100 nm spectral window was measured using a UV–vis–NIR spectrophotometer. The MPs at the bottom of the Eppendorf tube were redispersed in DI water and exposed to the output beam of a short‐pulsed OPO‐based laser tuned to 1064 nm optical wavelength, corresponding to a fluence of ≈24.2 mJ cm^−2^. After each exposure of 1, 2, and 3 min, the MPs dispersion was processed as described above to obtain the absorption spectra of the supernatants. In addition, based on the calculated amount of CuS loaded on the MPs, an equal concentration of free CuS NPs solution was subjected to the same process, and its spectra were measured as a control.

### Cell Viability Assay

CHO cells were cultured in DMEM supplemented with 10% FBS in a humidified incubator (Heracell 150, Thermo Fisher Science, USA) with a 5% CO2 environment at 37 °C. A 200 µL cell suspension with a cell amount of 3 × 10^4^ cells mL^−1^ was distributed into 96‐well plates and incubated for 24 h to allow attachment and proliferation. Subsequently, the cells were exposed to CuS NPs and PEG‐CuS NPs at concentrations of 0.54, 1.07, 1.61, 2.14, and 4.23 mм or CaCO_3_ MPs, CaCO_3_@CuS MPs, CaCO_3_@CuS/PDA MPs at concentration of 0, 2.5 × 10^6^, 5 × 10^6^, 7.5 × 10^6^, 1 × 10^7^, 2 × 10^7^ particles mL^−1^ for 24 additional hours. After the removal of the suspension medium, each well was washed with PBS. Then, 200 µL of 10% alamar blue in DMEM without FBS was added to each well and the cells were cultured for an additional 4 h. The fluorescence signal at an excitation wavelength of 540 nm and emission wavelength of 590 nm in each well was recorded by a microplate plate reader (Infinite 200 Pro, Tecan, Switzerland).

(2) $$\mathrm{Cell}\;\;\mathrm{viability}=\frac{\mathrm{Fluorescence}\;\mathrm{intensity}\;\mathrm{from}\;\mathrm{experimental}\;\mathrm{group}}{\mathrm{Fluorescence}\;\mathrm{intensity}\;\mathrm{from}\;\mathrm{control}\;\mathrm{group}}\times 100\%$$

### OA Tomographic Imaging of the Mouse Brain

OA imaging of the mouse brain was done with the above‐described spherical array, which was oriented downward inside a custom‐made water tank filled with degassed DI water. A central opening at the bottom of the tank was sealed with a polyethylene film with the mouse head placed under the film. A liquid light guide (Thorlabs, LLG3‐4Z) was inserted in a central aperture of the spherical array to direct the light beam from the OPO laser toward the imaged mouse. A sequence of OA images at 1064 nm wavelength was acquired over a 6 min duration (36 000 frames).

Female athymic nude mice (18 g, 4 weeks) were obtained from Envigo and housed in ventilated cages within a temperature‐controlled environment under a 12‐h dark/light cycle. The temperature was maintained at 21 ± 1 °C, with a relative humidity of 55 ± 10%. Pelleted food and water were provided ad libitum. For in vivo imaging, the mice (body weight: 20–25 g, age: 5–8 weeks) were anesthetized using isoflurane (5% v/v for induction and 1.5% for maintenance, Abbott, Cham, Switzerland) in an oxygen/air mixture (100/400 mL min^−1^). Data acquisition was done by securing the mouse's head using a custom‐designed stereotactic holder combined with a breathing mask, fixed onto the imaging platform. Continuous monitoring of blood oxygen saturation, heart rate, and body temperature (PhysioSuite, Kent Scientific, USA) was done during data acquisition, and the body temperature was maintained at ≈36 °C using a heating pad. Likewise, the temperature inside the water tank was maintained at 36 °C. To ensure proper acoustic coupling, ultrasound gel was applied to the scalp. A dose of 100 µL of a suspension of NPs (36.35 mм concentration, *n* = 4 mice) or MPs (2 × 10^8^ particles mL^−1^, *n* = 2) was administered through the tail vein. Following data acquisition, the mice were euthanized under deep anesthesia (5% isoflurane for 5 min) and subsequently decapitated. All in vivo experiments were performed in accordance with the Swiss Federal Act on Animal Protection and approved by the Cantonal Veterinary Office Zürich.

### Tracking Biodistribution and Pharmacokinetics of Nano‐ and Micro‐Particles with sSVOT

The schematic of the sSVOT scanner is described elsewhere.^[^ ^36^ ^]^ Briefly, the same spherical array described above was combined with a custom‐made fiber bundle (CeramOptec GmBH, Bonn, Germany) that bifurcates into five output fiber bundles, each placed at 40 mm distance from array's center. One fiber bundle was inserted into the central cavity of the array, while the other four arms were arranged around its circumference with the help of a custom‐designed holder. An OPO‐based Nd:YAG laser (Model: Spitlight 1200 OPO, Innolas GmbH, Krailling, Germany), operating at 10 Hz repetition rate, delivered pulses at 1064 nm wavelength through all five output fiber bundles. Each fiber output created a Gaussian illumination profile of 10 mm diameter at FWHM at the tissue surface, resulting in a ≈10 mm height and ≈31 mm arc length illumination pattern covering the entire width of the mouse.^[^ ^55^ ^]^ The animal was held in a fixed position inside a water tank with fore and hind paws secured to a custom‐designed animal holder.^[^ ^56^ ^]^ The spherical array together with output fiber bundles was scanned continuously on the back of the mouse along the vertical (z) direction. A single vertical sweep of the sSVOT scanner takes less than 2 s to cover the entire width of the mouse from the neck to tail region. The position of the spherical array was controlled with a motorized stage that can be translated in the vertical direction (RCP2‐RGD6c‐I‐56P‐4‐150‐P1‐S‐B, IAI Inc., Shizuoka Prefecture, Japan). The exact position of the array was monitored using a high‐resolution distance sensor (Keyence Deutschland GmbH, Neu‐Isenburg, Germany). The generated OA signals were digitized with the same DAQ unit, as described above. Whole‐body 3D images of the mice were rendered by compounding the individual volumetric frames acquired at each position of the array using the Icmax technique.^[^ ^36^ ^]^

### LOT Image Processing and Reconstruction

LOT imaging was performed over 36 000 frames following the MP injection. The acquired raw OA signals were initially band‐pass filtered between 0.1 and 6 MHz and a singular value decomposition (SVD) clutter filter was applied to effectively separate signal fluctuations attributed to absorbing particles in vascular flow from static signals originating from endogenous absorbers.^[^ ^22^ ^]^ The time‐lapse sequence of images was reconstructed using a GPU‐based back‐projection algorithm. Within each OA image volume, precise localization of individual MPs was achieved by identifying the local maxima positions (built‐in Matlab function “imregionalmax”) and computing correlation coefficients between small regions surrounding these maxima and a model point spread function (PSF) specific to the imaging setup. Tailoring the particle tracking approach to specific experimental parameters, an optimal algorithm was employed to trace the trajectories of these mobile MPs over consecutive frames, ultimately reconstructing the full spatiotemporal particle flow map.

### Ex Vivo H&E Staining

At 105 min post‐injection, *n* = 2 mice injected with MPs, *n* = 2 mice injected with NPs, and *n* = 1 mouse injected with PBS (control), were given a lethal dose of ketamine (75–100 mg kg^−1^), xylazine (10 mg kg^−1^), and acepromazine maleate (2‐3 mg kg^−1^). Liver, kidneys, brain, lungs, heart, and spleen were excised from the mice, immersed in 4% paraformaldehyde for 24 h, washed three times by PBS, dehydrated in gradient ethanol aqueous solution, and subsequently embedded in paraffin. Tissue sections from these organs were then subjected to histopathological analysis with hematoxylin‐eosin (H&E) staining according to the standard protocol. The samples were further studied using a conventional bright‐field microscope equipped with a color CMOS camera.

### Biosafety Study

The biosafety of the MPs was assessed in female Swiss mice injected i.v. with a single 100 µL dose of 1) PBS (*n* = 4, control group), 2) CaCO_3_@CuS/PDA MPs at a concentration of 2 × 10^8^ particles mL^−1^ (*n* = 4, high concentration group), and 3) 5 × 10^7^ particles mL^−1^ (*n* = 4, low concentration group). Animals were randomly assigned to the three groups and surveyed for 1 week, with regular (5 times a week) weight control. Blood samples were obtained, and hematological analysis was performed by means of a Mindray BC5000‐Vet analyzer at days 1, and 7 post‐injection. At the endpoint (7 days post‐injection), the blood was extracted for biochemical analysis.

## Conflict of Interest

The authors declare no conflict of interest.

## Supporting information



Supporting Information

Supplemental Movie 1

Supplemental Movie 2

Supplemental Movie 3

## Data Availability

The data that support the findings of this study are available from the corresponding author upon reasonable request.
