# Supplementary material for: Scalable Copper Sulfide Formulations for Super‐Resolution Optoacoustic Brain Imaging in the Second Near‐Infrared Window
Source: Small Methods. 2024 Oct 24;9(1):2400927. doi: 10.1002/smtd.202400927 (PMC11740951; doi:10.1002/smtd.202400927)
Supplement: Supplementary file 1 — Supporting Information [file SMTD-9-2400927-s002.docx]

Supporting Information

Scalable Copper Sulfide Formulations for Super-resolution Optoacoustic Brain Imaging in the Second Near-infrared Window

Lin Tang, Daniil Nozdriukhin, Sandeep Kumar Kalva, Quanyu Zhou, Çağla Özsoy, Shuxin Lyu, Michael Reiss, Anxo Vidal, Ana Torres, Xosé Luís Deán-Ben, * and Daniel Razansky *

**This PDF file includes:**

Figure S1 to S11

Table S1

Legends for movie S1 to S3

**Other Supplementary Materials for this manuscript include the following:**

Movie S1 to S3


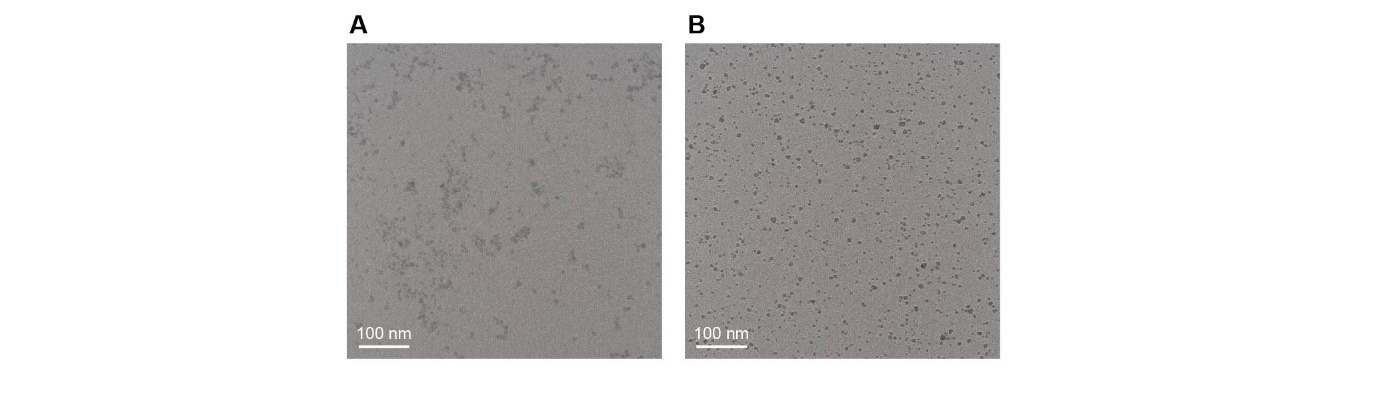


**Figure S1.** TEM microphotographs of A) CuS NPs and B) PEG-CuS NPs.


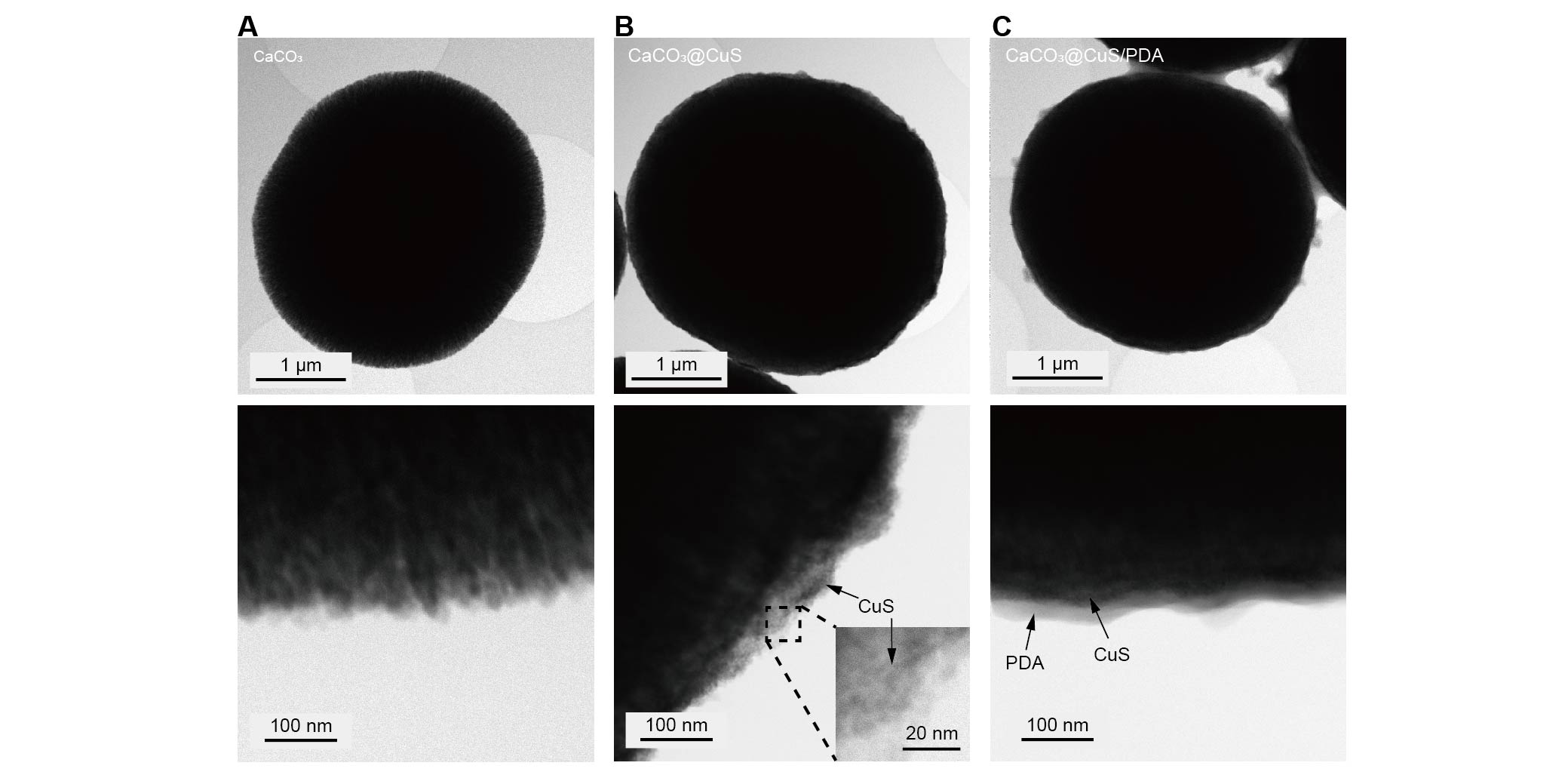


**Figure S2.** TEM microphotographs of A) CaCO_3_ MPs, B) CaCO_3_@CuS MPs, and C) CaCO_3_@CuS/PDA MPs.


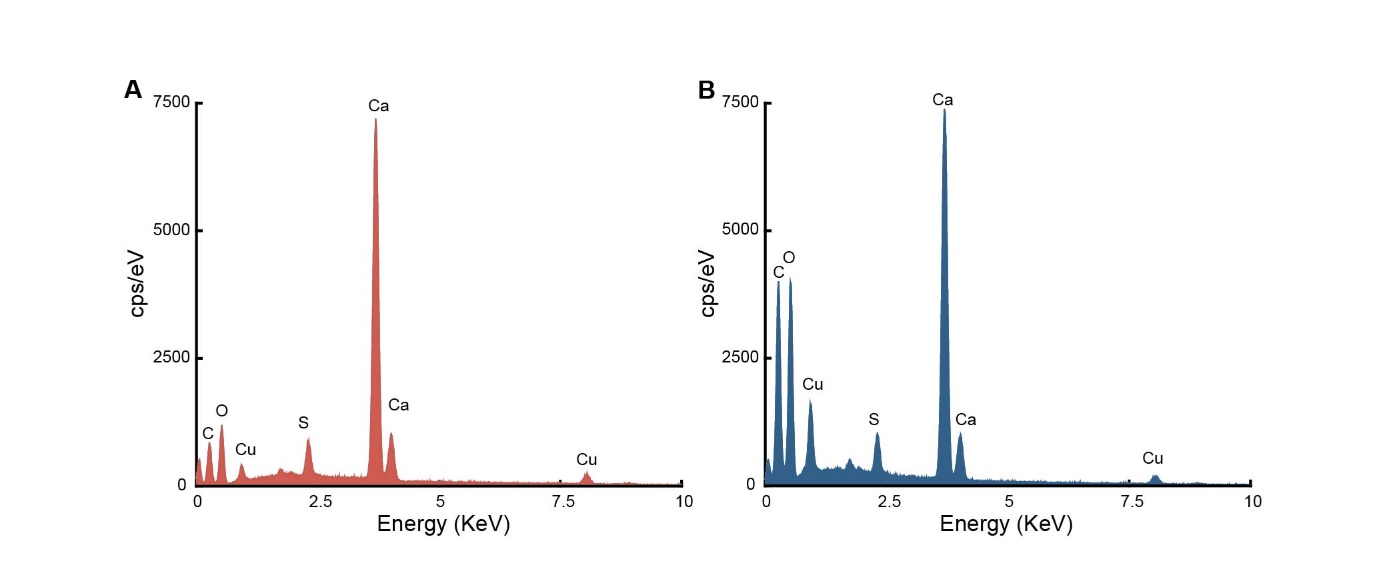


**Figure S3.** Point EDS spectra of A) CaCO_3_@CuS MPs and B) CaCO_3_@CuS/PDA MPs.


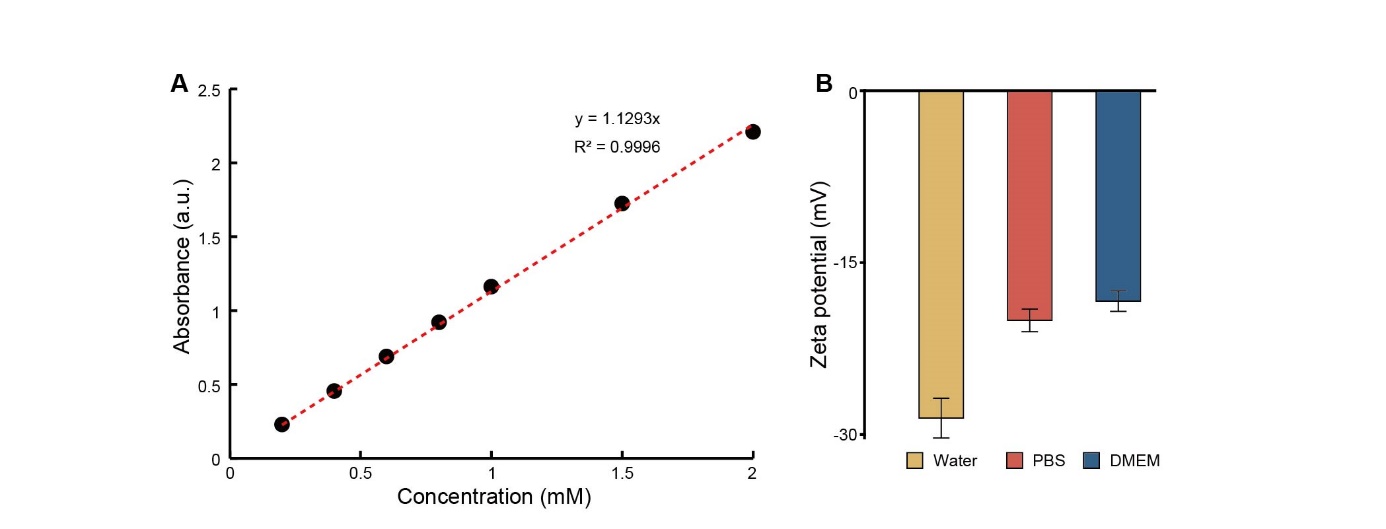


**Figure S4.** A) Standard linear calibration curve illustrating the relationship between the concentration of copper sulfide nanoparticles (CuS NPs), as measured by inductively coupled plasma optical emission spectrometry (ICP-OES), and the corresponding absorbance at 997 nm optical wavelength. B) Zeta potential values of CaCO_3_@CuS/PDA MPs dispersed in water, PBS, and DMEM (*n* = 3). Data was shown as mean ± SD.


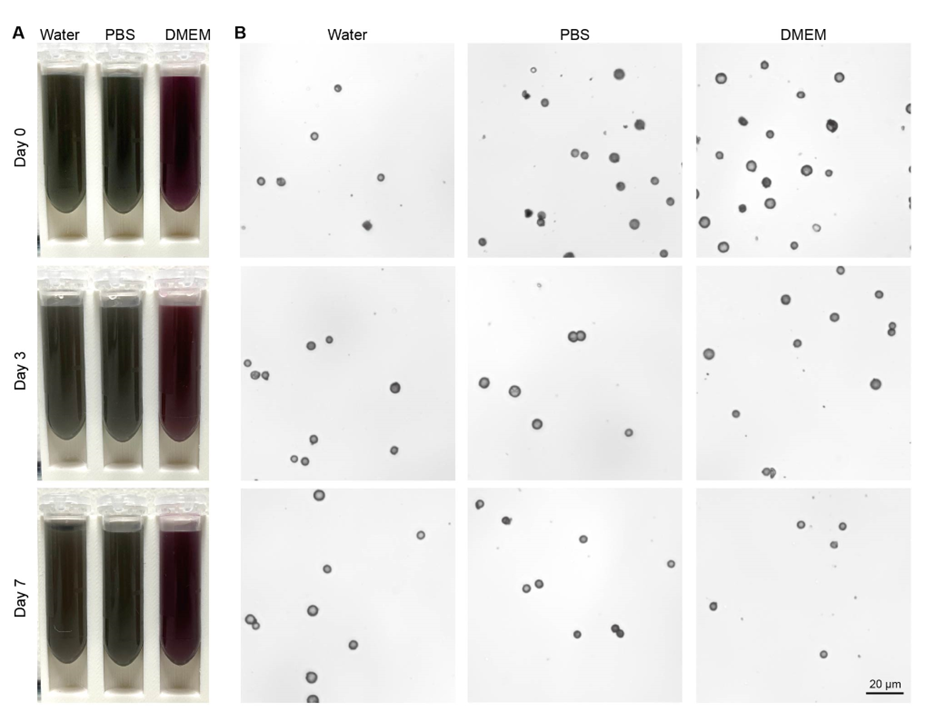


**Figure S5**. A) Digital photographs and B) bright-field microscope images of CaCO_3_@CuS/PDA MPs dispersed in water, PBS, and DMEM at different time points.


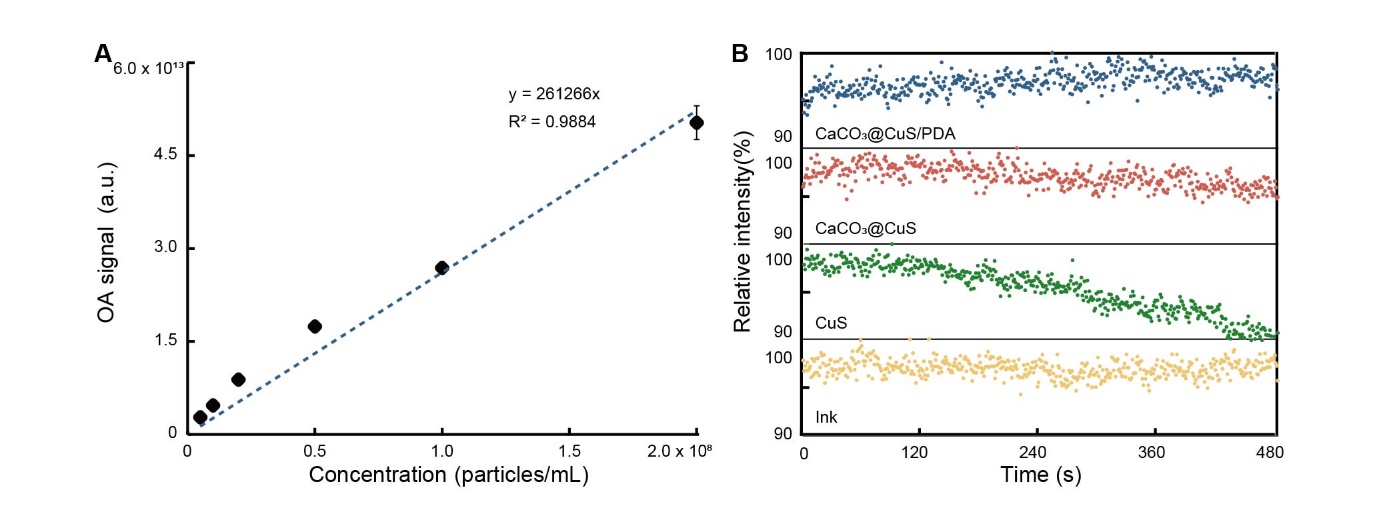


**Figure S6.** A) Optoacoustic signal excited at 1064 nm optical wavelength as a function of CaCO_3_@CuS MPs concentration. B) Optoacoustic signal of CuS NPs, CaCO_3_@CuS MPs, and CaCO_3_@CuS/PDA MPs exposed to a short-pulsed light beam at 1064 nm optical wavelength and ~24.2 mJ cm^-2^ fluence for 8 min. The equivalent ink signal is displayed as a reference.


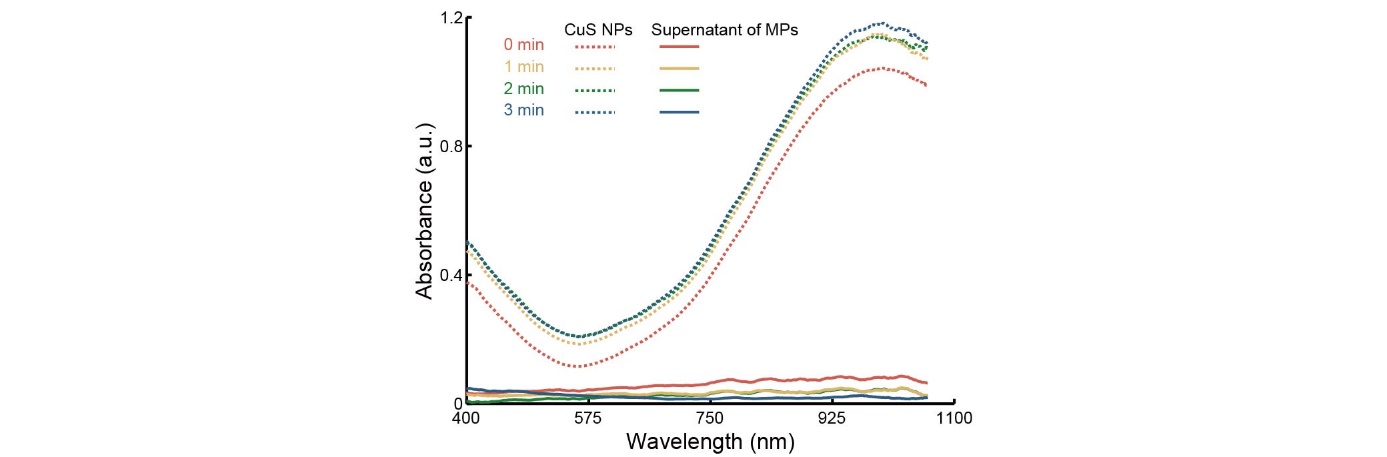


Figure S7. Absorption spectra of the upper solution of MPs (solid line) and equal amount of CuS NPs stock solution (dash line) after irradiation with a short-pulsed light beam at 1064 nm and ~24.2 mJ cm^-2^ fluence for 0, 1, 2, and 3 min.


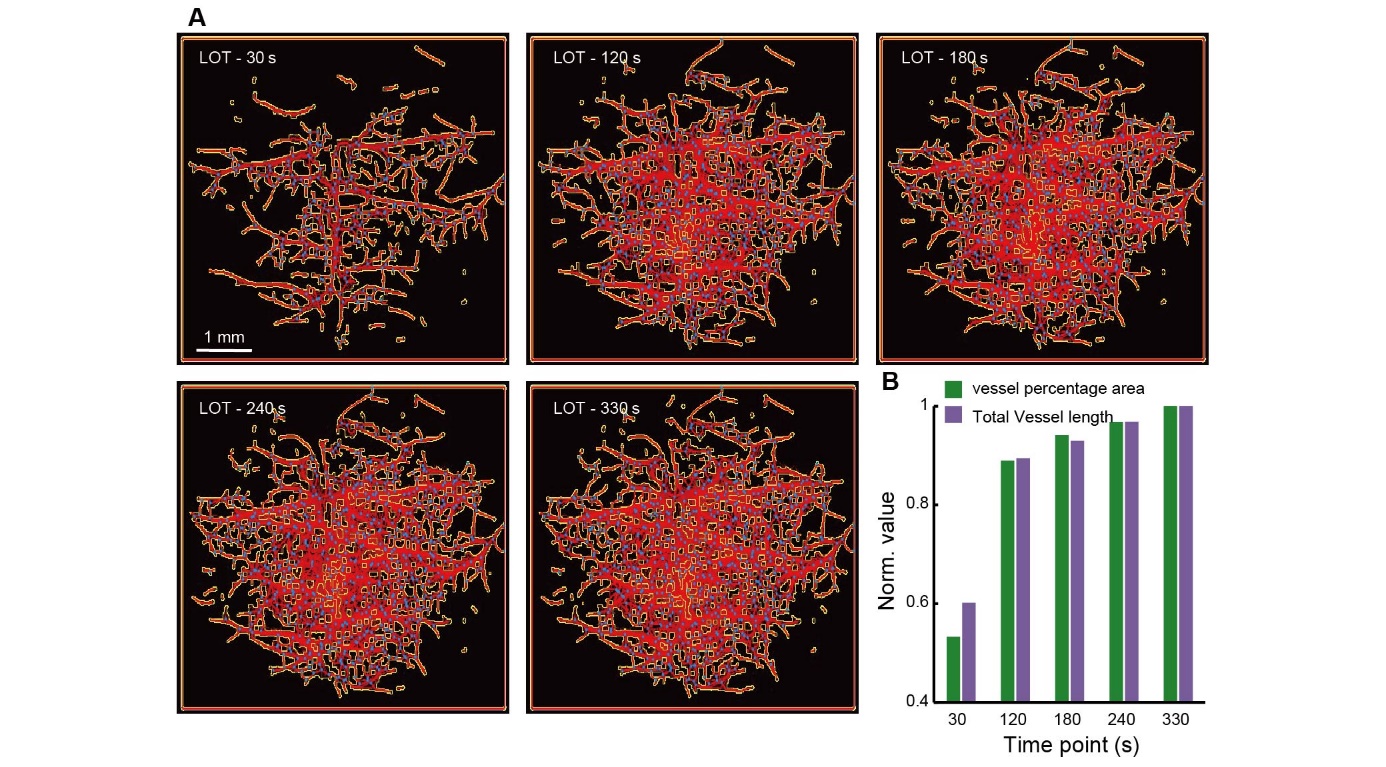


**Figure S8.** A) Vessels segmented from maximum intensity projections of the LOT data using AngioTool for various reconstruction periods (localization frame counts). B) Quantitative assessment of the vessel visibility as a function of reconstruction period.


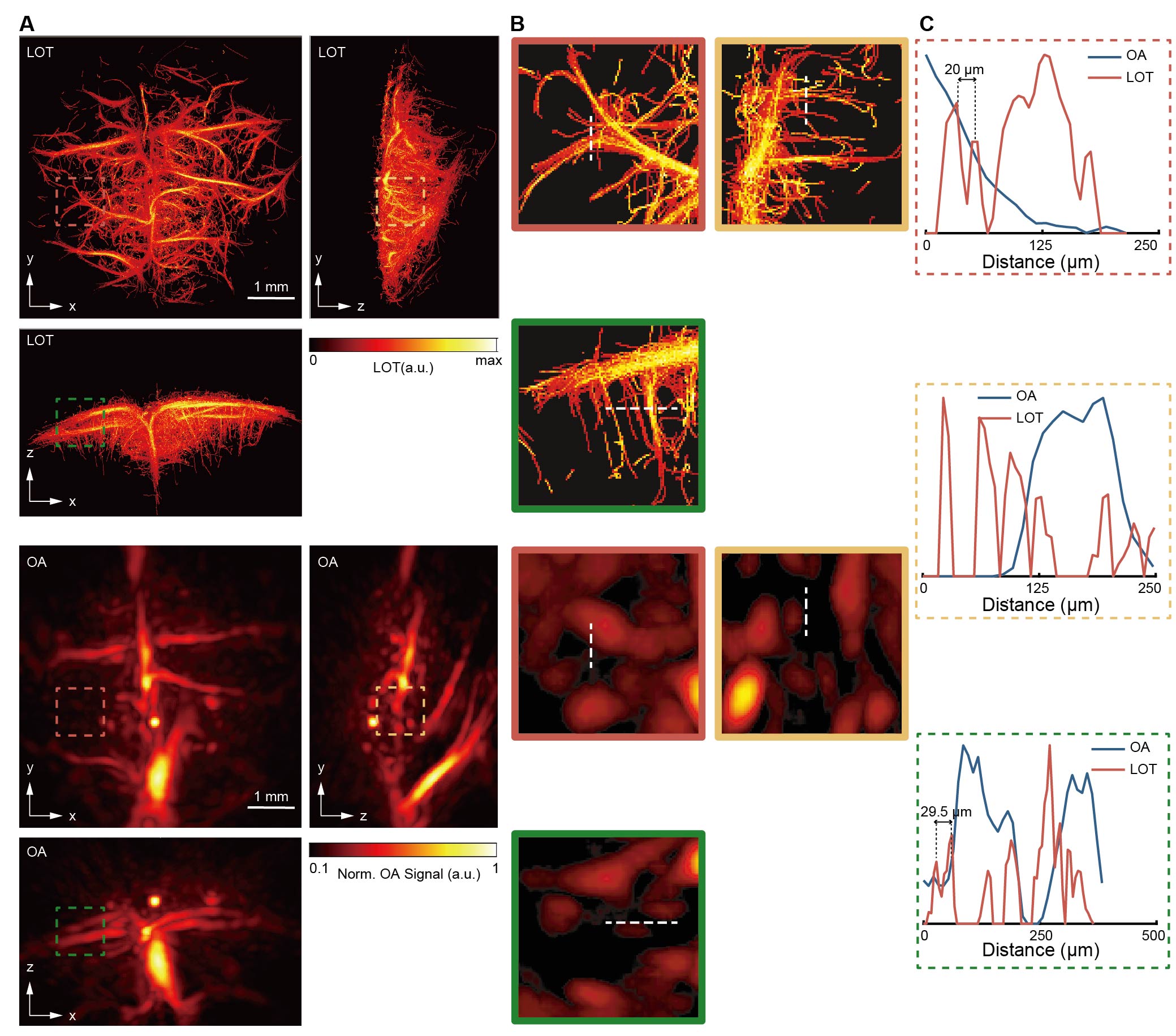
**Figure S9.** *In vivo* brain imaging with OA and LOT. A) Maximum intensity projections (MIPs) along the z, x, and y axes of the 3D images rendered with LOT and OA. B) Magnified views of the LOT and OA MIPs shown in panel A focusing on the regions within the square boxes; red - transverse plane, yellow - coronal plane, green - sagittal plane. C) Line profiles along the dashed white lines in the OA and LOT images from panel B.


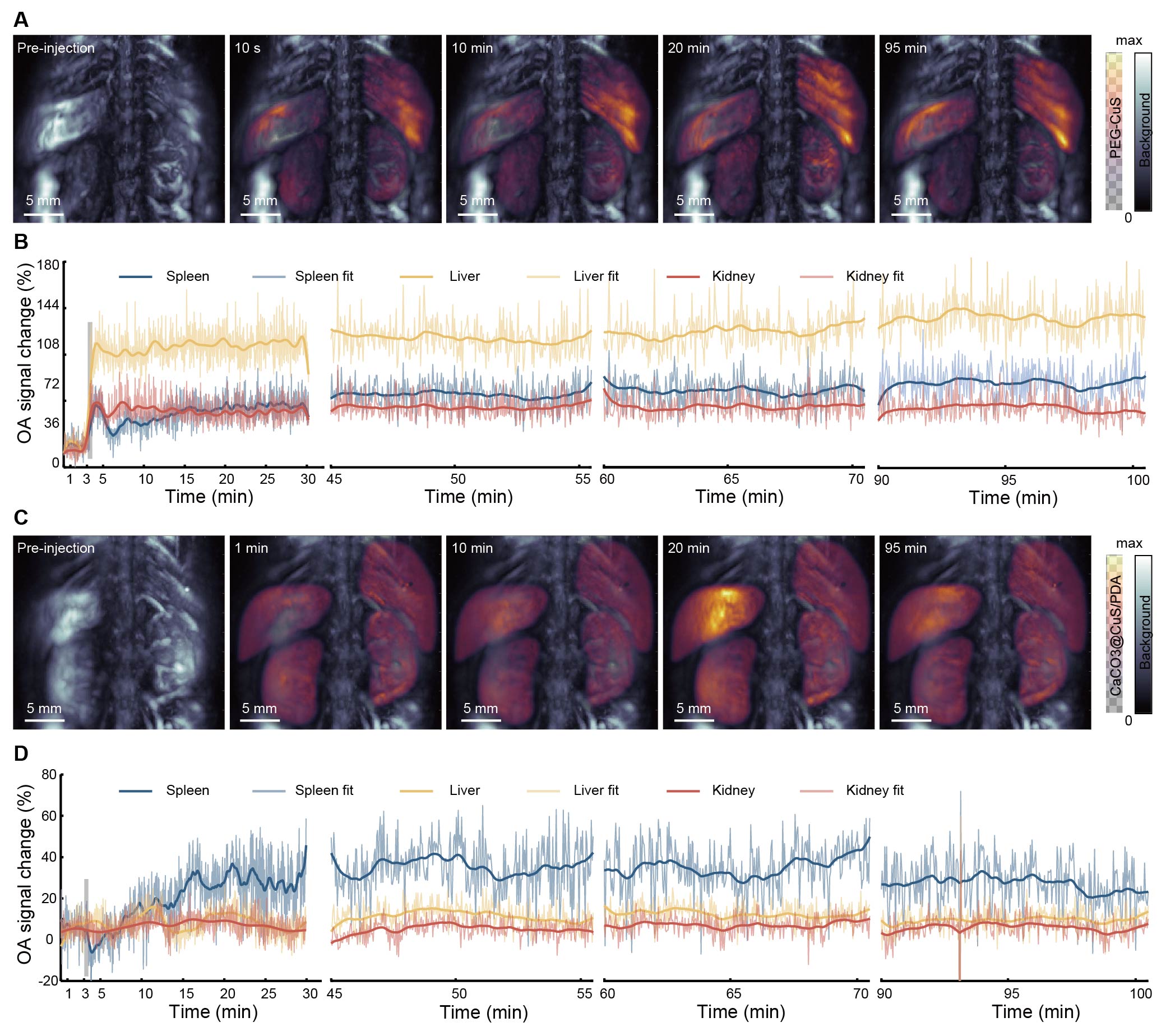


**Figure S10.** A) sSVOT images of a mouse back acquired at 1064 nm optical excitation wavelength over 100 min after the injection of PEG-CuS NPs and B) the corresponding organ accumulation curves. C) sSVOT images of a mouse back acquired at 1064 nm optical excitation wavelength over 100 min after the injection of CaCO_3_@CuS/PDA MPs and D) the corresponding organ accumulation curves. The color of the organs indicates relative signal change with respect to the pre-injection state.


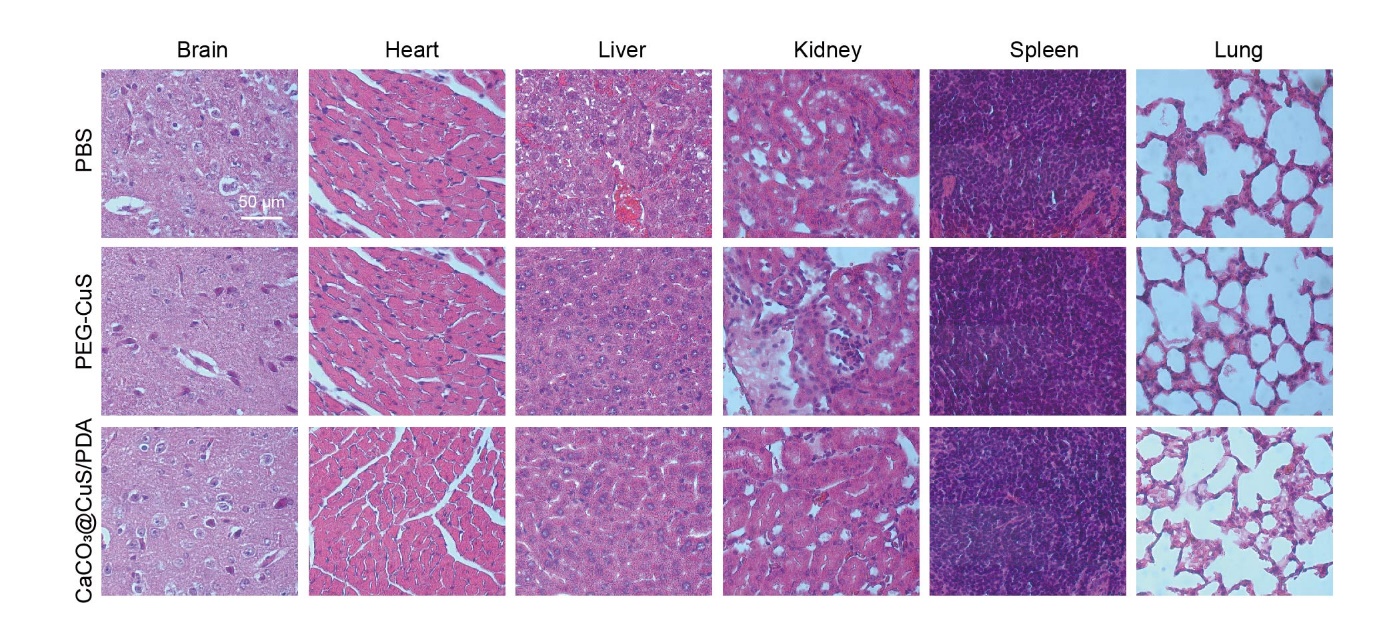


**Figure S11.** Results of hematoxylin & eosin (H&E) staining of slices from murine organs. No microparticles or pathological changes were detected.

**Table S1.** Hematology results of the control group of mice (injected with PBS) and the CaCO_3_@CuS/PDA-treated group of mice at 1- and 7-days post-injection.

| Parameter  (× 10^9^ L^-1^) | | PBS | CaCO_3_@CuS/PDA LC | CaCO_3_@CuS/PDA HC |
| --- | --- | --- | --- | --- |
| Day 1 | WBC | 5.345 ± 0.964 | 5.500 ± 0.508 | 7.048 ± 0.898 |
|  | Lym | 4.045 ± 0.641 | 4.450 ± 0.406 | 4.898 ± 0.641 |
|  | Neu | 0.745 ± 0.226 | 0.683 ± 0.204 | 1.295 ± 0.386 |
|  | Mon | 0.495 ± 0.212 | 0.303 ± 0.062 | 0.778 ± 0.211 |
|  | Eos | 0.060 ± 0.031 | 0.065 ± 0.019 | 0.083 ± 0.025 |
|  | Bas | 0.000 ± 0.000 | 0.000 ± 0.000 | 0.000 ± 0.000 |
| Day 7 | WBC | 6.075 ± 0.636 | 5.613 ± 0.426 | 7.895 ± 0.473 |
|  | Lym | 4.565 ± 0.640 | 4.215 ± 0.536 | 6.133 ± 0.849 |
|  | Neu | 0.955 ± 0.100 | 0.935 ± 0.250 | 1.053 ± 0.264 |
|  | Mon | 0.468 ± 0.070 | 0.393 ± 0.078 | 0.628 ± 0.209 |
|  | Eos | 0.085 ± 0.041 | 0.070 ± 0.021 | 0.083 ± 0.022 |
|  | Bas | 0.003 ± 0.005 | 0.000 ± 0.000 | 0.000 ± 0.000 |

LC: low concentration group, 5×10^7^ particles mL^-1^; HC: high concentration group, 2×10^8^ particles mL^-1^. Values are expressed as mean ± SD (*n* = 4).

SUPPLEMENTARY MOVIES

Supplementary Movie 1.

Real-time optoacoustic imaging of PEG-CuS nanoparticle perfusion in the murine brain.

Supplementary Movie 2.

Localization optoacoustic tomography image of murine brain vasculature based on CaCO_3_@CuS/PDA microparticle tracking.

Supplementary Movie 3.

Visualization of the flow of individual CaCO_3_@CuS/PDA microparticles from transverse, coronal and sagittal views. Differential optoacoustic (OA) image (green colormap) is superimposed to the LOT image (orange colormap).
